# Glucose Oxidase-Mediated Polymerization as a Platform for Dual-Mode Signal Amplification and Biodetection

**DOI:** 10.1002/bit.23101

**Published:** 2011-02-17

**Authors:** Brad J Berron, Leah M Johnson, Xiao Ba, Joshua D McCall, Nicholas J Alvey, Kristi S Anseth, Christopher N Bowman

**Affiliations:** 1 Department of Chemical and Biological Engineering, ECCH 111, UCB 424, University of ColoradoBoulder, Colorado 80309-0424; telephone: 303-492-3247; fax: 303 492 4341; e-mail: christopher.bowman@colorado.edu; 2 Howard Hughes Medical Institute, University of ColoradoBoulder, Colorado 80309

**Keywords:** biological detection, enzymatic polymerization, dual-mode amplification

## Abstract

We report the first use of a polymerization-based ELISA substrate solution employing enzymatically mediated radical polymerization as a dual-mode amplification strategy. Enzymes are selectively coupled to surfaces to generate radicals that subsequently lead to polymerization-based amplification (PBA) and biodetection. Sensitivity and amplification of the polymerization-based detection system were optimized in a microwell strip format using a biotinylated microwell surface with a glucose oxidase (GOx)–avidin conjugate. The immobilized GOx is used to initiate polymerization, enabling the detection of the biorecognition event visually or through the use of a plate reader. Assay response is compared to that of an enzymatic substrate utilizing nitroblue tetrazolium in a simplified assay using biotinylated wells. The polymerization substrate exhibits equivalent sensitivity (2 µg/mL of GOx-avidin) and over three times greater signal amplification than this traditional enzymatic substrate since each radical that is enzymatically generated leads to a large number of polymerization events. Enzyme-mediated polymerization proceeds in an ambient atmosphere without the need for external energy sources, which is an improvement upon previous PBA platforms. Substrate formulations are highly sensitive to both glucose and iron concentrations at the lowest enzyme concentrations. Increases in amplification time correspond to higher assay sensitivities with no increase in non-specific signal. Finally, the polymerization substrate generated a signal to noise ratio of 14 at the detection limit (156 ng/mL) in an assay of transforming growth factor-beta. Biotechnol. Bioeng. 2011; 108:1521–1528. © 2011 Wiley Periodicals, Inc.

## Background

Many biodetection platforms designed for resource limited environments are limited by inadequate sensitivity. As one significant example, recent flu test kits designed for rapid detection of the H1N1 virus lacked the required sensitivity to detect the levels of viral load in 10–70% of positive patients (Ginocchio et al., 2009), leading to a potential delay in treatment. Robust, rapid, and easily implementable biodetection systems possessing high sensitivity and easily discernable yes/no signals stand to positively impact personal and global health care.

This work develops a novel design for an enzyme substrate solution which utilizes polymerization for additional signal amplification in ELISA and other immunodetection applications. The ability to detect low concentrations of analyte in a biological sample is typically accomplished through either substrate or signal amplification. Substrate amplification methodologies such as PCR tend to be substrate specific and difficult to simply and cheaply deploy on a large scale. In signal amplification, most commonly, an enzymatic label is used to generate an observable signal from a limited number of biological interactions. For example, a tetramethylbenzidine (TMB) substrate with a horseradish peroxidase label has emerged as an effective platform for signal amplification (Holland et al., 1974; McKimmbreschkin, 1990). These ELISA platforms use horseradish peroxidase to catalyze the conversion of TMB to a blue reaction product, which is typically measured at 370 or 655 nm. The successful implementation of peroxidase-based amplification can be countered by the presence of endogenous peroxidases which generate non-specific responses (Boenisch, 2001). Additionally, the amplification generated by horseradish peroxidase is often limited by signal that results from non-specifically bound enzyme (Adams, 1992). There is no fundamental mechanism to limit the generation of signal from ultra-low concentrations of non-specifically bound enzyme.

The use of a glucose oxidase (GOx) label is one approach to overcome endogenous peroxidases in mammalian analytes (Suffin et al., 1979). In one such commercialized substrate solution, GOx mediates amplification through the conversion of water-soluble nitroblue tetrazolium (NBT) into an insoluble formazan precipitate (Suffin et al., 1979). The NBT approach is also limited in total amplification by non-specifically generated signal (Adams, 1992).

In contrast, polymerization-based amplification (PBA) is a signal amplification approach in which each radical that is generated participates in a large number of propagation events that couple monomers and other labels to the biodetection event, leading to rapid, large, and specific amplification (Sikes et al., 2008, 2009). PBA utilizes selective binding of a polymerization initiator conjugate to achieve specificity of the polymerization initiator localization (Hansen et al., 2008b; Lou et al., 2005). This initiator bound sample is then exposed to a monomer solution and excited with an energy source appropriate to the selected initiator, for example, light for a photoinitiator. Once initiation commences, the initiating radical species locally consume inhibitor species and rapidly form a polymer film on the scale of tens of nanometers to several microns, depending on the target concentration, the initiator, and the monomer (Johnson et al., 2009a; Lou and He, 2006; Qian and He, 2009). PBA has been demonstrated to detect as few as 60 surface-bound biomarkers (Hansen et al., 2008a). For these highly sensitive applications, the PBA approach currently necessitates both a high-intensity light source and nearly complete elimination of oxygen from the monomer formulation (Avens et al., 2008). These requirements are both readily achieved in a clinical setting with appropriate facilities and instrumentation, but these requirements preclude the use of PBA in some resource limited biodetection applications (Urdea et al., 2006).

Here, we present a novel approach towards signal amplification offering dual-mode amplification in a single substrate solution, utilizing the combination of enzymatic catalysis and PBA. We utilize GOx that is selectively bound to a target and subsequently capable of rapidly catalyzing the polymerization of a fluorescent polymer film. Iwata et al. established the basic approach towards GOx-mediated polymerization while developing a novel detachable balloon catheter device (Iwata et al., 1991, 1992). In this approach, the GOx-mediated reaction of β-d-glucose to δ-d-gluconolactone reduces the enzyme (Eq. 1). The subsequent oxidation by molecular oxygen regenerates the enzyme and produces hydrogen peroxide (Eq. 2). The hydrogen peroxide is then converted to hydroxyl radicals through Fenton's Chemistry (Eq. 3) where the hydroxyl radicals are then capable of initiating conventional radical polymerization, each one propagating through many monomers prior to terminating.


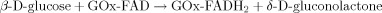
(1)


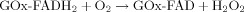
(2)


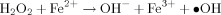
(3)


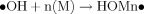
(4)

**Figure 1:**
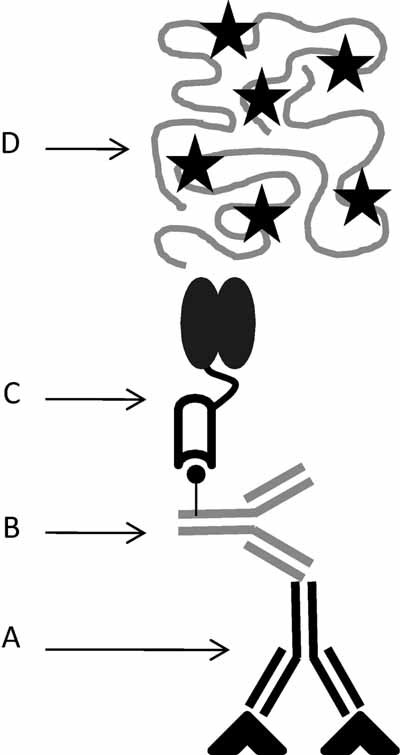
Schematic approach to polymerization-enhanced enzyme substrate solution for biodetection. **A**: Antigen is recognized by primary antibody. **B**: Incubation with a biotinylated secondary antibody imparts biotin functionality. **C**: Sample is incubated with a glucose oxidase–avidin conjugate, and the resulting surface is decorated with specifically bound glucose oxidase. **D**: The sample is then incubated in a monomer solution containing poly(ethylene glycol) diacrylate, hydroxyethyl acrylate, rhodamine B-methacrylate (indicated by stars), glucose, FeSO_4_, and MES, resulting in the formation of a fluorescent polymer. Polymer may be qualitatively observed visually or a plate reader may be employed for quantitation.

Johnson et al. (2009b) have previously demonstrated GOx-mediated polymerization to be a non-cytotoxic approach toward cellular encapsulation. Here, we build on these previous implementations of GOx-mediated polymerizations for applications in biodetection. A GOx-avidin D conjugate (GOx-Av) is used to selectively immobilize the enzyme and the subsequent polymerization events in wells expressing a specific interaction between biotin and GOx-Av (Fig. 1). This novel approach enables the use of GOx to generate many hydrogen peroxide molecules, while each subsequently generated radical leads to the polymerization of many monomers prior to termination. The fluorescent monomer unit copolymerizes directly into the forming polymer matrix and thus provides a covalently coupled marker through which to characterize and interpret the polymerization. Our combination of both enzymatic and polymerization amplification constitutes a novel, two-mode amplification system that is more robust, is capable of greater specific signal intensity, and potentially increases assay sensitivity.

This enzymatic approach eliminates the equipment resources (e.g., light sources) previously required by photoinitiated PBA (Hansen et al., 2008b; Johnson et al., 2009a; Sikes et al., 2008), allowing the use of PBA in locations a light source and purge gas system are impractical. Additionally, this system uses GOx, an enzyme that has been exploited in blood glucose testing applications (Boland et al., 2001) and possesses exceptional thermal stability (Vasileva and Godjevargova, 2004). In all, the fundamental construct of this technology is promising for highly sensitive biological testing in resource-limited environments.

## Materials and Methods

### Polymerization Substrate Solution Optimization

Biotin Coated Clear 8-Well Strip Plates (Pierce, Rockfield, IL) were rinsed for 15 min with 0.1% bovine serum albumin in phosphate-buffered saline. Well contents were aspirated, and the wells were incubated for 30 min with the appropriate dilution of the GOx-Av (Vector Laboratories, Burlingame, CA, *Aspergillus niger* source) in 0.1% bovine serum albumin and phosphate-buffered saline. An additional negative control (NC) consisted of 1 mg/mL unconjugated GOx (Sigma–Aldrich, St. Louis, MO) in 0.1% bovine serum albumin in phosphate-buffered saline. Wells were rinsed with 0.1% bovine serum albumin in phosphate-buffered saline, and presented with a mixture of FeSO_4_ (Sigma–Aldrich), β-d-glucose (Sigma–Aldrich), 15 wt.% poly(ethylene glycol) diacrylate (PEGDA, *M*_n_ ∼ 575 Da; Sigma–Aldrich), 20 wt.% hydroxyethyl acrylate (HEA; Sigma–Aldrich), 35 µM methacryloxyethyl thiocarbamoyl rhodamine B (Polysciences, Warrington, PA, excitation/emission: 548 nm/570 nm) and 20 mM 2-(*N*-morpholino)ethanesulfonic acid buffer stabilized at pH 4.5 (Teknova, Hollister, CA) in water. The polymerization step proceeded protected from light in ambient atmosphere and at ambient temperature. In argon-purged studies, the well strips were filled with monomer and placed in a continuously purged quart sized plastic bag. In pH studies, the monomer solution pH was adjusted from 4.5 through the addition of a 1.0 M sodium hydroxide solution (Fisher, Waltham, MA). Polymerization was halted by removing unreacted monomer with a water rinse. Fluorescence was immediately measured using a Wallac-Victor^2^ 1420 plate reader (Perkin Elmer, Waltham, MA) excited at 550 nm and measured at 590 nm. Each condition was repeated three times, and the reported error corresponds to the standard deviation of the measured values.

### Standard Polymerization Substrate Solution

For clarity, the standard polymerization substrate solution is provided here as 250 µM FeSO_4_, 512 mM β-d-glucose, 15 wt.% poly(ethylene glycol) diacrylate (*M*_n_ ∼ 575 Da), 20 wt.% HEA, 35 µM methacryloxyethyl thiocarbamoyl rhodamine B, and 20 mM 2-(*N*-morpholino)ethanesulfonic acid buffer stabilized at pH 4.5 in water.

### Fourier Transform Infrared Kinetic Studies

Polymerizaiton kinetics were monitored using a Nicolet 750 Magna Fourier Transform Infrared (FTIR) Spectrometer using a nitrogen cooled MCT detector and a white light source. The presence of the acrylate functionality was measured by the absorption of the first overtone of acrylate C–H stretching in the near IR region between 6,212 and 6,150 cm^−1^ (Johnson et al., 2009b; Weyer and Lo, 2006). Polymerization commenced with the addition of the GOx-Av to a mixture of FeSO_4_, β-d-glucose, 15 wt% poly(ethylene glycol) diacrylate, 20 wt% HEA, and 20 mM 2-(*N*-morpholino)ethanesulfonic acid buffer stabilized at pH 4.5 in water. Samples were mixed and placed in a glass, 1 mm sample compartment and placed in the instrument for measurement (Berchtold et al., 2001). All polymerization kinetics studies were performed without inert gas purging and at ambient temperature. Acrylate concentration was linearly interpolated from the initial baseline acrylate peak area corresponding to 2.24 M with the absence of an observable acrylate peak area corresponding to complete consumption of the acrylate. Polymerization rate (*R*_p_) was determined from the average rate of change of the acrylate concentration between 5% and 15% conversion. Each condition was repeated three times, and the reported error corresponds to standard deviation of the measured values.

### Detection of TGF-β

Transforming growth factor-beta (TGF-β) was used to demonstrate protein detection. NUNC Maxisorp plates were incubated with dilutions of TGF-β in phosphate buffer overnight at 4°C. Plates were then rinsed five times and blocked with 0.1% bovine serum albumin in phosphate buffer for 4 h at 4°C. Plates were then rinsed five times and incubated in a 1:100 dilution of mouse antibodies against TGF-β in 0.1% bovine serum albumin in phosphate buffer overnight. Plates were then rinsed five times with 0.1% bovine serum albumin in phosphate buffer and incubated for 1 h in 1:200 dilution of biotinylated anti-mouse secondary antibody in 0.1% bovine serum albumin in phosphate buffer. Plates were rinsed five times and incubated with 100 µg/mL GOx-Av in 0.1% bovine serum albumin in phosphate buffer for 30 min. Wells were rinsed three times with 0.1% bovine serum albumin in phosphate-buffered saline, and presented with the standard polymerization substrate solution for 4 h.

## Results and Discussion

### Polymerization Substrate Solution Optimization

The successful implementation of the sequential enzymatic and PBA necessitates an understanding of the complex roles that each component plays in controlling and facilitating the reactions necessary for sensitive, specific biodetection. For example, as indicated, the iron is necessary to decompose the hydrogen peroxide into the peroxy radicals that initiate the reaction while also participating to facilitate termination of the radicals during the polymerization. Oxygen has a similar dual role, and thus, it is necessary to understand first the impact of these various components on both the radical generation and polymerization reactions. Throughout these studies, specific amplification was verified through the use of two distinct negative controls. Wells not incubated with the GOx-Av served as a negative control against non-enzymatically initiated polymerization, and wells incubated with a concentrated solution of unconjugated GOx served as a control against non-specific enzyme adsorption.

**Figure 2:**
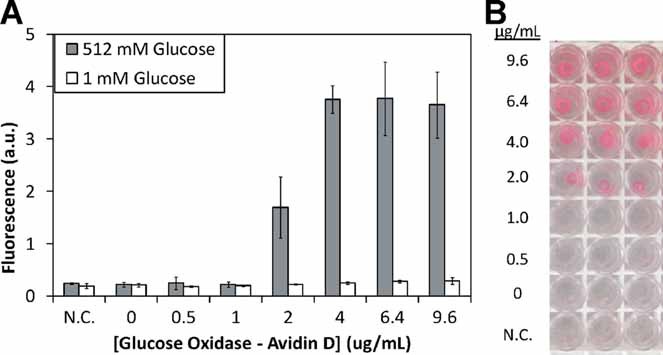
Fluorescent assay response to glucose variation. **A**: Wells were incubated with indicated glucose oxidase–avidin dilutions. A negative control well was incubated with 1 mg/mL of unconjugated glucose oxidase. Wells were then incubated in a polymerization substrate solution consist of the indicated glucose concentration, 15 wt.% PEGDA, 20 wt.% HEA, 35 µM rhodamine B-methacrylate, 250 µM FeSO_4_, and 20 mM MES pH = 4.5 for 30 min. **B**: Digital photograph of three replicates of fluorescent assay with 512 mM glucose.

The oxidation of β-d-glucose to δ-d-gluconolatone by GOx is followed by the regeneration of the GOx FAD co-factor (i.e., FAD oxidation) and H_2_O_2_ production. Regeneration of the enzyme is critical to the cyclic peroxide formation, and thus, the enzymatic amplification mode of this enzyme-mediated PBA. As an initial demonstration of the capabilities of this technique, eight-well strips surface-functionalized with biotin were first incubated for 30 min with varying dilutions of a GOx-Av which were subsequently exposed to substrate solutions containing varying glucose amounts. The substrate solution consisted of either 512 mM glucose and 15 wt.% PEGDA, 20 wt.% HEA, 35 µM rhodamine B-methacrylate, 250 µM FeSO_4_, 20 mM MES pH = 4.5 (standard polymerization substrate) or the standard polymerization substrate using only 1 mM glucose. The monomer/glucose solutions were in contact with the well for 30 min under an ambient atmosphere before the wells were rinsed and fluorescence output from each well was measured. A digital camera image of the visual 512 mM glucose assay response and a compilation of the fluorescence measurements are presented in Figure 2. The monomer mixture containing 512 mM glucose exhibited high signal to noise (15 ± 3), and a sensitivity of 2 µg/mL of GOx-Av. Over the measured range of GOx-Av concentrations, the 1 mM glucose solution failed to polymerize, and positive signal intensities were indistinguishable from that of the NC. These results both demonstrate the appropriateness of this technique for biodetection and also indicate the critical role of glucose in the GOx-mediated polymerization amplification scheme (Johnson et al., 2009b).

**Figure 3:**
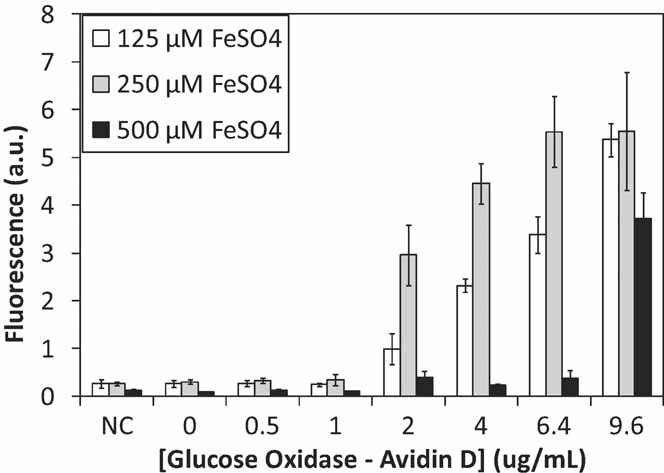
Fluorescent assay response to FeSO_4_ variation. Wells were incubated with indicated glucose oxidase–avidin dilutions. A negative control well was incubated with 1 mg/mL of unconjugated glucose oxidase. Wells were then incubated in polymerization substrate solution consist of the indicated FeSO_4_ concentration, 15 wt.% PEGDA, 20 wt.% HEA, 35 µM rhodamine B-methacrylate, 512 mM glucose, and 20 mM MES pH = 4.5 and for 30 min.

As previously discussed, iron plays a direct role in the conversion of enzymatically generated hydrogen peroxide to hydroxy radicals, which are then capable of initiating polymerization. As in the assay of glucose dependence, biotinylated eight-well strips were sequentially presented with varying concentrations of GOx-Av and polymerization substrate solutions containing low (125 µM), intermediate (250 µM), or high (500 µM) concentrations of FeSO_4_. After removing unreacted material, the fluorescence of each well was analyzed, and the results are presented in Figure 3. The sensitivity of the assay was 4, 2, and 9.6 µg/mL of Gox-Av for FeSO_4_ concentrations of 125, 250, and 500 µM, respectively. At high GOx-Av concentrations (9.6 µg/mL), all iron species exhibit a large positive signal (signal/noise > 20) while at low GOx-Av concentrations, an optimum Fe^2+^ concentration is observed. The reduction of fluorescence intensity for the high iron conditions results from a reduced final monomer conversion, a previously observed phenomenon (Johnson et al., 2009b) associated with the inhibitory reactions (Eqs. 5 and 6) resulting from both ferric and ferrous species in solution (Dainton and Seaman, 1959; Walling, 1975).


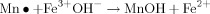
(5)


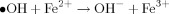
(6)

The GOx-Av concentration-dependent behavior is also consistent with infrared measurements of the polymerization kinetics (Supplemental Fig. S2) which demonstrate exaggerated sensitivity to iron only at low GOx-Av concentrations.

Both the GOx and hydrogen peroxide decomposition reactions are known to be dependent on the pH of the reaction media, and studies were performed to understand the importance of possible pH variations on the ability for GOx to facilitate biodetection by polymerization. As such, polymerization substrate solutions were adjusted to a pH of 4.5, 5.5, 6.5, and 7.5 with 1.0 M sodium hydroxide. The assay was carried out as described previously after contact with 9.6 µg/mL GOx-Av, and a positive signal was only generated at pH 4.5. The lack of polymerization under more basic conditions is consistent with the rapid, base-catalyzed oxidation of Fe^2+^ to Fe^3+^ (Tabakova et al., 1992). This reaction is strongly dependent on pH, and only the Fe^2+^ species facilitates polymerization (Eq. 3) while the Fe^3+^ species acts as a strong polymerization inhibitor (Eq. 5).

Many sources indicate optimal GOx activity at a pH of approximately 5.5 (Auses et al., 1975; Hanft and Koehler, 2006; Nicol and Duke, 1966). While we have previously demonstrated the capacity for GOx-mediated radical polymerization at pH levels between 4.5 and 7.4 (Johnson et al., 2009b), this success was achieved only in the presence of much higher concentrations of GOx (equivalent to 60 µg/mL of GOx-Av directly mixed into the monomer solution). As the measurements here incorporate only the surface-immobilized enzyme, the actual enzyme concentration present in this system is many orders of magnitude lower than that of the previous studies. A higher GOx concentration increases the overall initiation rate as needed to overcome inhibitory reactions. These findings are consistent with the present observation of an optimal FeSO_4_ concentration only at low GOx concentrations, while no optimum is observed at higher GOx concentrations (*vide infra*).

The limitation of narrow pH range could be alleviated through the incorporation of more enzyme per binding event, in an analogous approach to polymeric enzyme detection (Klonoski et al., 2010). The resulting increase in enzyme concentration would increase the initiation rate, and potentially enable the use of a wider range of pH conditions. Additionally, the use of a polymeric enzyme detection approach would likely improve the detection limit of our polymerization substrate solution another 10- to 100-fold (Klonoski et al., 2010).

**Figure 4:**
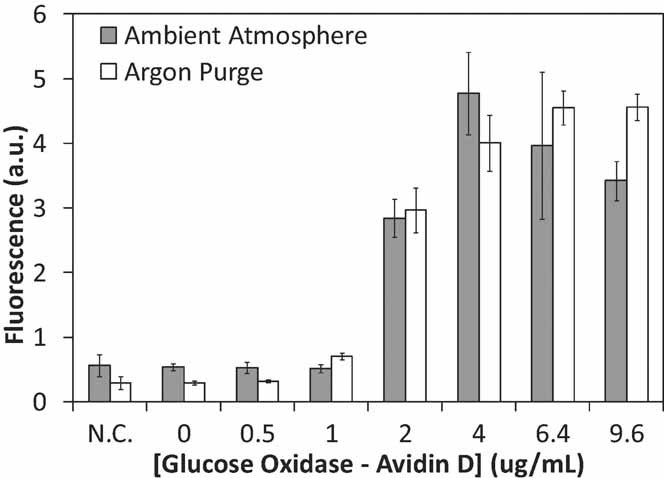
Fluorescent assay dependence on polymerization atmosphere. Wells were incubated with the indicated glucose oxidase–avidin dilutions. A negative control well was incubated with 1 mg/mL of unconjugated glucose oxidase. Wells were then incubated in a polymerization substrate solution consist of 15 wt.% PEGDA, 20 wt.% HEA, 35 µM rhodamine B-methacrylate, 512 mM glucose, 20 mM MES pH = 4.5, and 250 µM FeSO_4_ for 30 min in the indicated atmosphere.

One of the greatest potential benefits of the proposed methodology is the opportunity to perform polymerization-based biodetection assays rapidly at ambient conditions. Clearly, overcoming the classical limitation of oxygen inhibition in conventional radical polymerizations is critical to realizing this benefit. Oxygen's inhibitory role in radical polymerization schemes arises through the reaction of molecular oxygen with an active radical chain, resulting in a radical species that is incapable of further propagation. This inhibitory effect of oxygen on radical polymerizations is the rationale for inert gas purging of polymerization systems. Other approaches to overcoming oxygen inhibition have also been demonstrated, including the rapid generation of large numbers of radicals to locally consume oxygen (Decker and Jenkins, 1985). Here, oxygen no longer acts solely as an inhibitor since in this amplification scheme, molecular oxygen serves as a reactant that is converted to hydrogen peroxide in the oxidation of the enzyme. Ultimately, this assay relies on both the consumption of oxygen by GOx (Eq. 2) as well as the generation of a large number of initiating hydroxyl radicals (Eqs. 1–3) to overcome the inhibitory effects of molecular oxygen. As the complete purging of oxygen will prohibit polymerization (Iwata et al., 1991), we qualitatively assessed the effect of oxygen on the system by comparing the proposed standard assay format which takes place in the ambient atmosphere to an assay format where the well strips are prepared in the ambient environment, and then incubated in an argon purged chamber for 30 min with the monomer/glucose solution. As shown in Figure 4, there was no observed improvement in assay sensitivity (2 µg/mL) or signal strength with the partial purging of oxygen from the system. The lack of purge effect is indicative of the consumption of oxygen being much faster than the rate of diffusion of oxygen back into the ambient atmosphere system. Intuitively, the oxygen is initially consumed through either GOx or an inhibitory reaction with the radicals, and while the oxygen concentration is high, relatively few propagation events take place prior to termination by the oxygen species. Once the oxygen concentration is sufficiently low, initiation rates are lower, but many more propagation events take place for each radical prior to termination.

**Figure 5:**
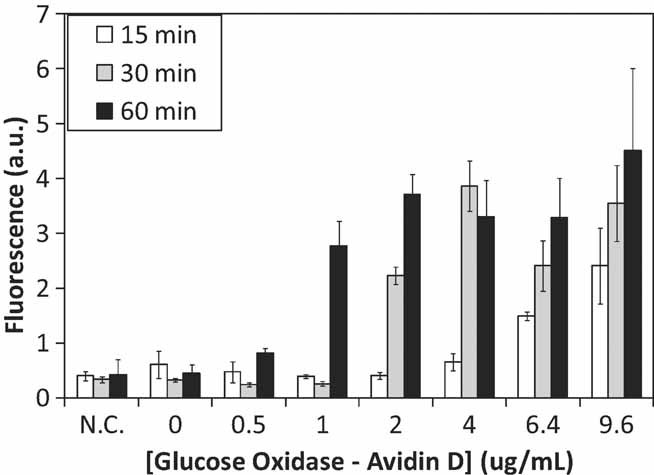
Fluorescent assay dependence on polymerization time. Wells were incubated with the indicated glucose oxidase–avidin dilutions. A negative control well was incubated with 1 mg/mL of unconjugated glucose oxidase. Wells were incubated in a polymerization substrate solution consist of 15 wt.% PEGDA, 20 wt.% HEA, 35 µM rhodamine B-methacrylate, 512 mM glucose, 20 mM MES pH = 4.5, and 250 µM FeSO_4_.

**Figure 6:**
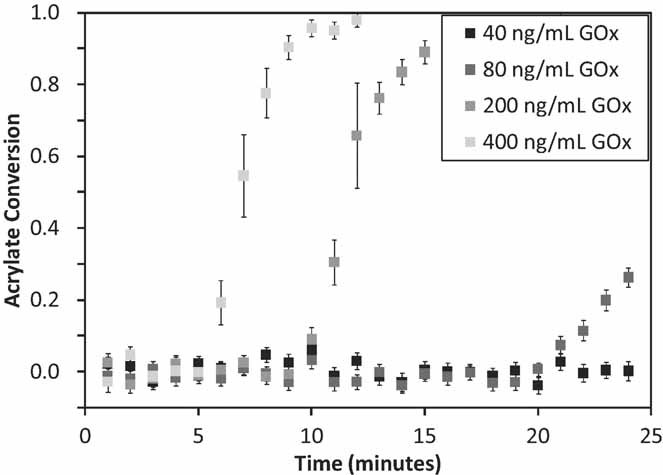
Induction time prior to polymerization for direct mixing of solution phase glucose oxidase. Representative acrylate conversion versus time data as determined by real-time Fourier-transform infrared spectroscopy. The polymerization substrate solution was consist of 15 wt.% PEGDA, 20 wt.% HEA, 35 µM rhodamine B-methacrylate, 512 mM glucose, 20 mM MES pH = 4.5, and 250 µM FeSO_4_.

As indicated in Figure 5, an increase in polymerization time resulted in an increase the overall sensitivity of the assay. The detection limits for 15, 30, and 60 min amplification times were 6.4, 2, and 1 µg/mL, respectively. This provides an opportunity to tune the incubation time to selectively amplify protein levels above a threshold value. The time-dependent increase in sensitivity is consistent with the time dependence of the polymerization induction time (Fig. 6).

Induction time represents the period of time prior to bulk polymerization during which an initiation system generates sufficient radicals to overcome any inhibitory reactions. Figure 6 shows real-time conversion of acrylate monomer, as measured by Fourier-transform infrared spectroscopy in samples where ultra-low concentrations of GOx were mixed directly into polymerization substrate solutions. Samples were prepared with 400, 200, 80, and 40 ng/mL concentrations of GOx-Av dispersed in the standard polymerization substrate solution. Acrylate conversion data shows for every twofold decrease in initiator concentration, there is an approximate twofold increase in induction time. This agreement between time and initiation scaling supports the supposition that the polymerization induction time is a major underlying factor determining the ability to generate a positive signal from PBA.

Uniquely, the increase in the amplification time did not increase the non-specific signal (Fig. 5). Many enzymatic amplification schemes are challenged by an increase in background signal at longer incubation times, limiting their capacity for amplification (Adams, 1992). One unique benefit of the polymerization induction time is that it translates into a complete lack of amplification from non-specifically adsorbed enzymes on practical assay time scales, limiting the possibility for false positives. In all, the polymerization induction time enables a clear distinction between specific and non-specific signal. Increased polymerization time increases the sensitivity of the assay, by allowing sufficient time for the initiation system to consume polymerization inhibitors.

### Comparison of Polymerization Substrate to Traditional Enzymatic Substrate

**Figure 7:**
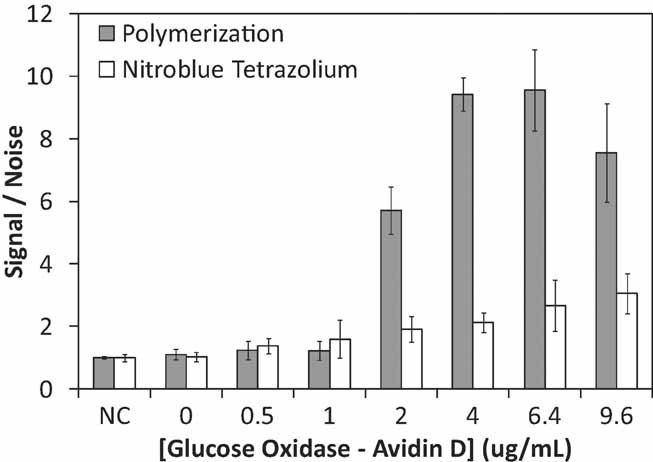
Comparison to commercial nitroblue tetrazolium assay. Assay response normalized to non-specific signal negative control (NC) for enzymatic polymerization-based amplification and commercial nitroblue tetrazolium assay at various glucose oxidase–avidin dilutions. A NC well was incubated with 1 mg/mL of unconjugated glucose oxidase. For polymerization-based amplification, wells were incubated in a polymerization substrate solution consist of 15 wt.% PEGDA, 20 wt.% HEA, 35 µM rhodamine B-methacrylate, 512 mM glucose, 20 mM MES pH = 4.5, and 250 µM FeSO_4_ for 30 min. Nitroblue tetrazolium system was amplified for 30 min per manufacturer's recommendations.

We contrasted the performance of our polymerization substrate system with that of a commercially available, NBT substrate for GOx (Fig. 7). Both sets of biotinylated well strips were subjected to identical blocking, incubation, and rinsing steps prior to amplification using the individual substrate systems. The polymerization-based system was amplified for 30 min in the standard polymerization substrate, while the NBT system was amplified per the manufacturer's directions for 30 min. Signal levels for each system were normalized to that of a NC well incubated with 1 mg/mL unconjugated GOx (negatively control condition). The detection limit (*P* < 0.05) was 2 µg/mL for both substrate solutions. Qualitatively, the polymerization substrate has a sharp increase in signal at the detection limit, where the NBT substrate exhibits the expected incremental increase in signal with higher enzyme concentration. One major distinction between the two approaches is that the signal to noise ratio for the polymerization approach (∼10) is much greater than that of the NBT assay (∼3). This behavior is attributed to both the dual mode amplification (enzymatic and polymerization) as well as non-specific signal being suppressed by long polymerization induction times.

### Polymerization Substrate Detection of TGF-β

**Figure 8:**
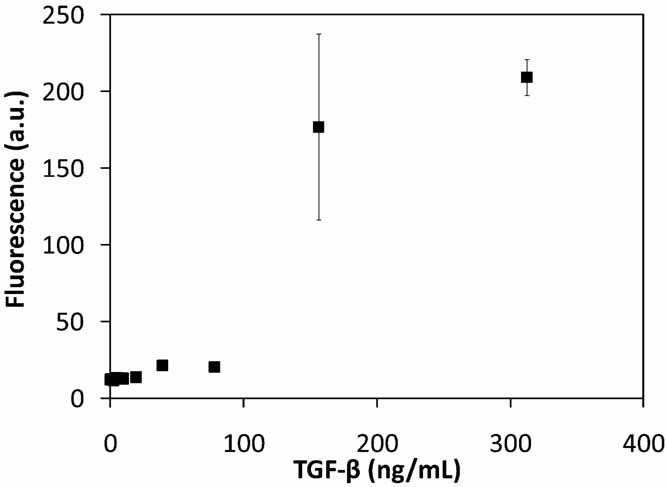
Polymerization substrate detection of TGF-β. Plates were sequentially incubated with TGF-β dilutions, mouse antibodies against TGF-β, biotinylated anti-mouse secondaries, and a glucose oxidase–avidin conjugate. Wells were then incubated in a polymerization substrate solution consist of 15 wt.% PEGDA, 20 wt.% HEA, 35 µM rhodamine B-methacrylate, 512 mM glucose, 20 mM MES pH = 4.5, and 250 µM FeSO_4_ for 4 h.

The polymerization-enhanced substrate was demonstrated through the detection of TGF-β. High-binding plates were incubated with dilutions of TGF-β in phosphate buffer overnight, and subsequently blocked, incubated with primary antibodies against TGF-β, biotinylated secondary antibodies, and a GOx-avidin conjugate at 100 µg/mL. Wells were rinsed and presented with the standard polymerization substrate solution for 4 h. After rinsing away unreacted substrate solution, well fluorescence was measured, and compiled in Figure 8. The polymerization enhanced substrate system possesses only specific amplification of the target protein. At TGF-β concentrations >156 ng/mL, there is a large signal which is readily distinguished from wells at lower concentrations (signal to noise ∼15).

## Conclusions

Enzyme-mediated polymerization was demonstrated to achieve comparable sensitivity, with greater signal amplification and signal to noise than commercial NBT amplification schemes. The enzyme-mediated polymerization system is strongly dependent on glucose, where higher glucose concentrations promoted a greater fluorescent signal. The initiation and inhibition roles of FeSO_4_ resulted in an optimal concentration for maximum sensitivity and amplification. Despite the well-documented polymerization inhibition by oxygen, there was not a detrimental effect of oxygen in the ambient atmosphere on this amplification system. Increases in total amplification time increased the overall assay sensitivity, without the typical corresponding increase in non-specific signal. Polymerization induction time is proposed as a governing condition on the ability to amplify a given biorecognition event. The large signal in conjunction with the negligible non-specific signal of this enzyme-mediated polymerization system is promising for applications in high sensitivity, qualitative response diagnostics in resource-limited settings.
