# Supplementary material for: Glucose Oxidase-Mediated Polymerization as a Platform for Dual-Mode Signal Amplification and Biodetection
Source: Biotechnol Bioeng. 2011 Feb 17;108(7):1521–8. doi: 10.1002/bit.23101 (PMC3098304; doi:10.1002/bit.23101)
Supplement: Supplementary file 1 [file bit0108-1521-SD1.pdf]

Supporting Information:

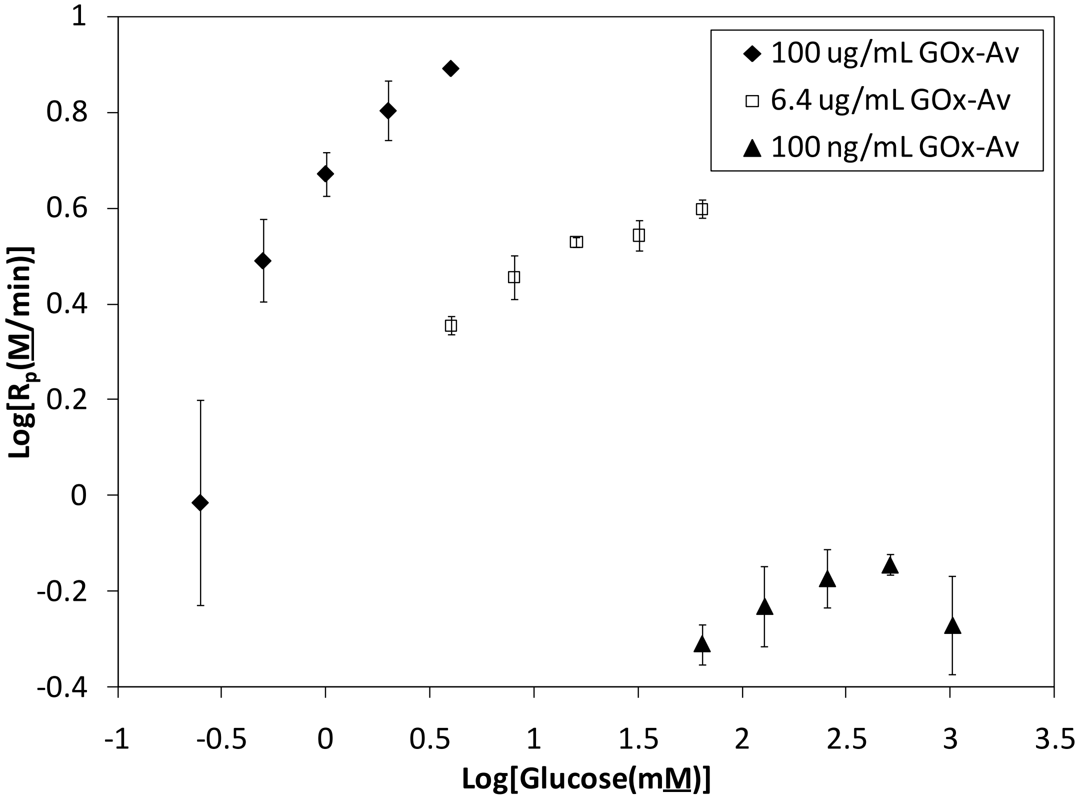

Figure S1 – Polymerization kinetics response to glucose variation. Polymerization rate dependence on β-D-glucose concentration for glucose oxidase enzyme concentrations of 100 µg/mL, 6.4 µg/mL and 400 ng/mL. Data collected using FTIR at 6175 cm^{-1} at room temperature using a monomer mixture containing 250 µM $FeSO_4$, 15 wt % PEGDA, 20 wt % HEA, 20 mM MES buffer in water. Error bars correspond to one standard deviation.

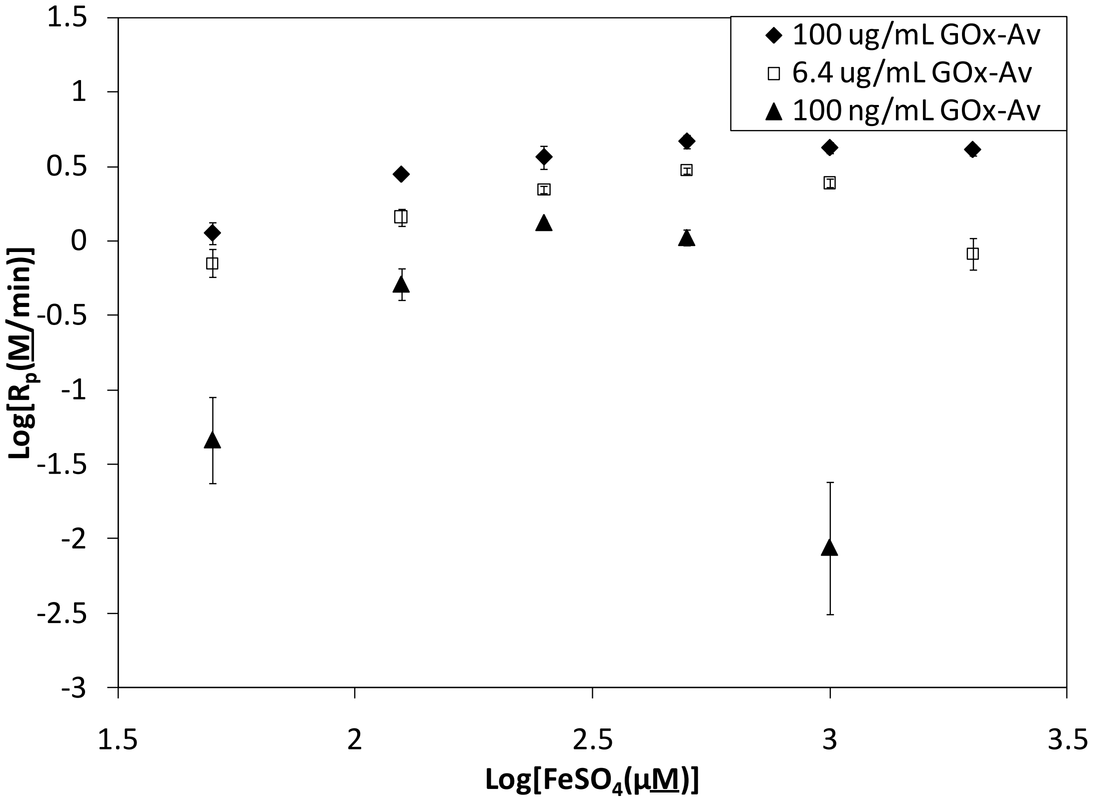

Figure S2 – Polymerization kinetics response to $FeSO_4$ variation. Polymerization rate dependence on [$FeSO_4$] for glucose oxidase enzyme concentrations of 100 µg/mL, 6.4 µg/mL and 400 ng/mL using glucose concentrations of 1 mM, 4 mM, and 512 mM, respectively . Data collected using FTIR at 6175 cm^{-1} at room temperature using a monomer mixture containing 15 wt % PEGDA, 20 wt % HEA, 20 mM MES buffer in water. Error bars correspond to one standard deviation.
